# The clinical efficacy analysis of ultrasound-assisted minimally invasive treatment for Jakob Type II humeral lateral condyle fractures in children

**DOI:** 10.3389/fped.2026.1643996

**Published:** 2026-06-08

**Authors:** Yunlong Liu, Sheng Ding

**Affiliations:** Department of Pediatric Surgery, The Affiliated Women and Children’s Hospital of Ningbo University, Ningbo, Zhejiang, China

**Keywords:** arthrography, humeral epicondyle fracture, Jakob, Kirschner wire, ultrasound

## Abstract

**Objective:**

This study explores the clinical efficacy of ultrasound-assisted minimally invasive treatment for Jakob Type II humeral lateral condyle fractures in children.

**Methods:**

A retrospective analysis was conducted on children with Jakob Type II humeral lateral condyle fractures who received treatment at our hospital between January 2021 and December 2024. Based on the surgical procedure, the patients were divided into two groups: the Ultrasound combined with x-ray arthrography-guided closed reduction and percutaneous pin fixation group (UA-CRPP) and the Open reduction percutaneous pin fixation group (ORPP). The UA-CRPP group underwent ultrasound-assisted closed reduction and internal fixation for Jakob Type II humeral lateral condyle fractures, with arthrography to assess the articular cartilage surface. The ORPP group underwent open reduction and Kirschner wire fixation for Jakob Type II humeral lateral condyle fractures. Demographic data, surgical time, clinical outcomes, complications, and radiographic data were recorded.

**Results:**

A total of 57 patients were included in both groups, with 37 males and 20 females. There were no significant differences between the two groups in terms of gender, age, weight, time from injury to surgery, follow-up time, injury side, or complications such as pin tract infection, deep infection, or intraoperative blood loss(*P* > 0.05). No cases of nonunion, refracture, or nerve injury were observed in either group. The surgical time and hospital stay were shorter in the UA-CRPP group compared to the ORPP group (*P* < 0.05), and the radiological union time of fracture was shorter in the UA-CRPP group (*P* < 0.05).

**Conclusion:**

Ultrasound-assisted closed reduction and internal fixation is a feasible and effective treatment option for children with Jakob Type II humeral lateral condyle fractures. Compared with ORPP, it has similar functional effects, but its advantages lie in less invasive, shorter surgical time, and lower complication rate.

## 1 Background

Among distal humeral fractures in children, lateral condyle fractures are relatively common, with an incidence rate second only to supracondylar fractures, accounting for approximately 12%–20% (1). According to the Jakob classification system (2), humeral lateral condyle fractures can be divided into three types: Type I: the articular surface remains intact, the cartilage hinge is continuous, and the fracture displacement is not obvious; Type II: the articular surface is ruptured, the cartilage hinge is disrupted, and the fracture fragments are displaced; Type III: the lateral condyle is completely displaced, and the fracture fragments also experience rotational displacement. For Type I fractures, conservative treatments such as cast immobilization are commonly used; Type III fractures typically require open reduction surgery; however, the treatment plan for Type II fractures remains controversial, with different opinions in the medical community. Lateral condyle fractures often involve the epiphysis, and improper management can lead to complications such as elbow deformities and traumatic arthritis, severely affecting the child's limb function development (3–5). Accurate assessment of fracture stability is critical for treatment planning and prognosis evaluation. Traditionally, joint arthrography has been an important method for evaluating the stability of such fractures, as it clearly demonstrates the integrity of the articular surface, providing a basis for surgical planning (6). However, arthrography cannot directly visualize injuries to the articular cartilage hinge, making it unreliable for predicting fracture stability (3). In recent years, ultrasound examination, with its advantages of being non-invasive, radiation-free, and capable of dynamic observation, has been increasingly used in the assessment of pediatric humeral lateral condyle fractures. Ultrasound can provide real-time imaging of fracture displacement, cartilage hinge continuity, and surrounding soft tissue damage, and it allows for repeated monitoring to dynamically assess treatment progress (7, 8). Nevertheless, musculoskeletal ultrasound is associated with a steep learning curve and technical challenges, including notable inter- and intra-observer variability (9–11). Therefore, this study aims to explore the clinical efficacy of ultrasound and joint arthrography in the treatment of Jakob Type II humeral lateral condyle fractures in children, aiming to leverage the advantages of both techniques. The goal is to provide more reliable evidence for clinical assessment and treatment, optimize treatment strategies, and improve the prognosis for affected children.

**Figure 1:**
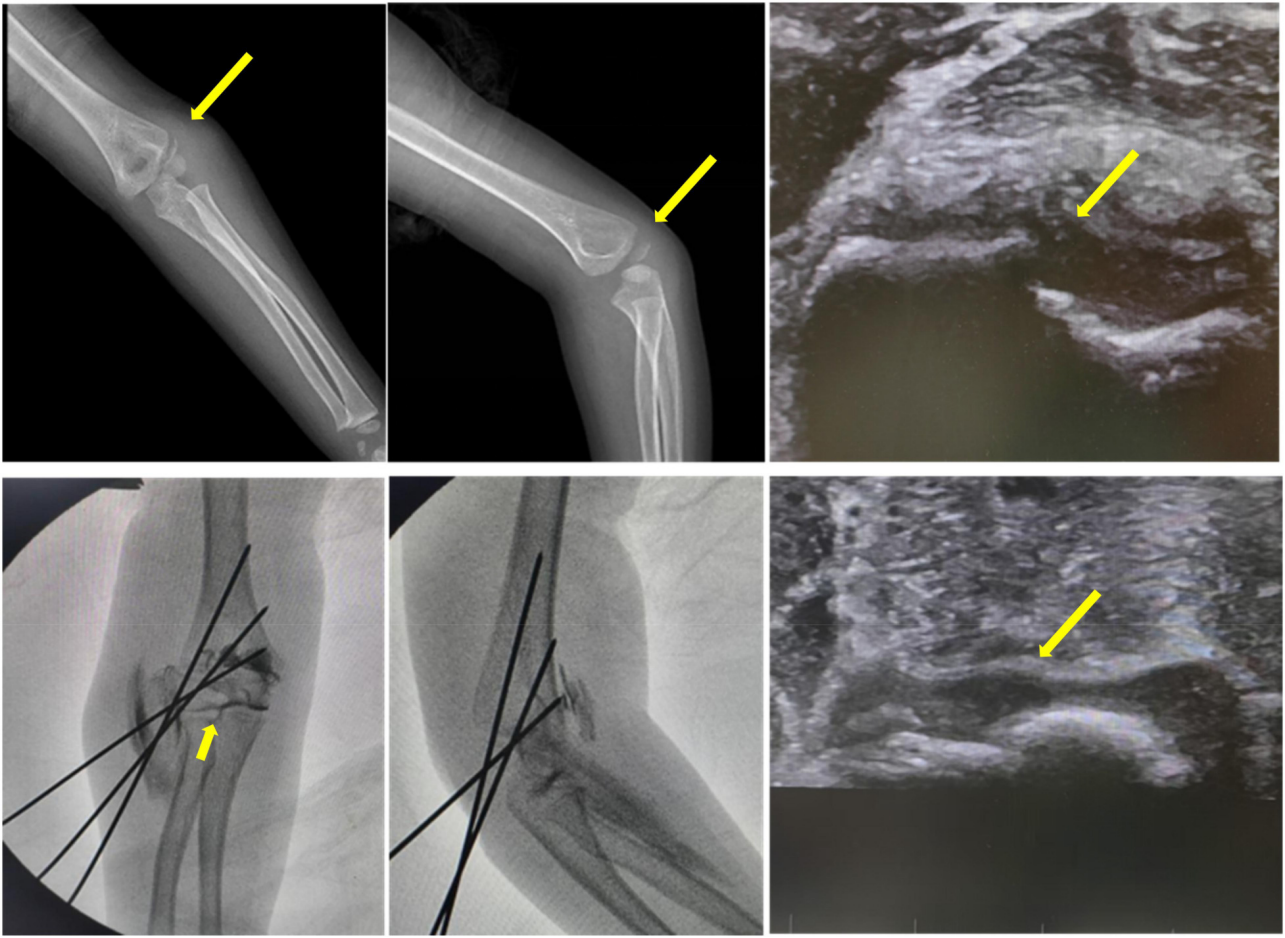
A 6-year-old boy with jakob type II humeral lateral condyle fracture. Preoperative radiographs showed displaced fracture fragment at the lateral humeral condyle. Intraoperative ultrasound demonstrated disruption of the cartilage hinge, with satisfactory reduction of fracture fragment and a smooth articular surface achieved after reduction. Postoperative arthrography following closed reduction and percutaneous pinning (CRPP) revealed adequate reduction of fracture fragment, an even articular surface without step-off.

## 2 Methods

### 2.1 Clinical and radiographs evaluation

A retrospective analysis was conducted on children with Jakob Type II humeral lateral condyle fractures who received treatment at our hospital from January 2021 to December 2024. Based on the surgical procedure, the patients were divided into two groups: the UA-CRPP group and the ORPP group. Demographic data, surgical time, clinical outcomes, complications, and imaging data were recorded. Inclusion criteria: (1) Diagnosis of humeral lateral condyle fracture; (2) Fracture classified as Jakob Type II. Exclusion criteria: (1) Open fractures; (2) Patients with pathological fractures, neuromuscular disorders, metabolic diseases, old humeral lateral condyle fractures, or concomitant fractures in other parts of the upper limb; (3) Patients with follow-up periods of less than 5 months or incomplete medical records. Informed consent was obtained from the parents, and the study was approved by the institutional ethics review board.

In total, 57 children with Jakob Type II humeral lateral condyle fractures were included in the study, with 37 males and 20 females. The average age was 5 ± 1.99 years, with 21 cases in the UA-CRPP group and 36 cases in the ORPP group. All patients were diagnosed preoperatively with x-rays (anterior-posterior and lateral views) or CT scans of the elbow joint. CT scans were not routine for Jakob classification assessment but were used only when individual children did not cooperate with x-ray examination; 2 cases in our series utilized CT. In the ORPP group, 36 cases underwent open reduction and Kirschner wire (K-wire) fixation, with x-ray used intraoperatively to assess reduction; in the UA-CRPP group, 21 cases underwent closed reduction and K-wire fixation, assisted by ultrasound for reduction and x-ray-guided joint contrast to evaluate the reduction. Musculoskeletal ultrasound was performed using a high-frequency linear array probe. Inter-observer consistency was maintained through regular training sessions. All ultrasound examinations were performed by the same two senior pediatric orthopedic surgeons (with over 5 years of experience in musculoskeletal ultrasound) to ensure consistency in the evaluation and decision-making process throughout the reduction procedure. Arthrography served dual purposes: confirming anatomical reduction of articular surface and assessing cartilage hinge continuity.

During follow-up, the Flynn elbow function evaluation was recorded at the last follow-up visit, along with complications. The comparison between the two groups included demographic data, surgical time, intraoperative blood loss, length of hospital stay, radiological union time of fracture, elbow function outcomes, and complications such as incision or pin tract infections, fracture re-displacement, and deformity healing. Deformity healing primarily referred to lateral epicondylar prominence, “fish-tail” deformity of the distal humerus, avascular necrosis of the fracture fragment, and non-union of the fracture.

### 2.2 Surgical treatment

The surgeries were performed by highly experienced senior physicians. In the UA-CRPP group, a tourniquet was not used, whereas in the ORPP group, a tourniquet was routinely applied to the upper limb. Musculoskeletal ultrasound was utilized, with the probe positioned at the anterior elbow to assess the disruption of the distal humeral joint cartilage, dynamically monitoring the degree of fracture displacement. Additionally, the anterior and lateral aspects of the elbow joint were examined to evaluate the sagittal and coronal displacement of the distal humerus.

The fracture fragment were manually reduced, and ultrasound was repeatedly used during the reduction process to monitor the reduction quality. Once reduction was satisfactory, 2–3 Kirschner wires (1.6–2.0 mm in diameter) were percutaneously inserted for internal fixation. The C-arm was used to evaluate the reduction and fixation of the fracture fragments. A posterior approach from the affected elbow side was used to inject 1 mL of Iohexol into the joint. After flexing and extending the elbow, x-rays were taken in both the anterior-posterior and lateral views to verify the reduction of the fracture fragment and the alignment of the joint cartilage surface, ensuring continuity and no step-offs in the joint surface, confirming good fracture reduction. Excessive K-wire tails were removed (Figure 1).

Postoperatively, a long-arm plaster cast was applied with the elbow in a neutral position. x-ray films were checked the day after surgery, and then every two weeks, until the fracture showed radiographic healing. The plaster cast and Kirschner wires were removed once healing was confirmed. Elbow function was assessed according to the Flynn grading system during the follow-up.

### 2.3 Statistical analysis

The statistical analysis was performed using SPSS 23.0 software. For normally distributed continuous variables, the data were presented as Mean ± SD, and the comparison between groups was made using the independent samples t-test. For non-normally distributed continuous variables, data were expressed as median (interquartile range), and group comparisons were conducted using the Mann–Whitney U test. Categorical data were expressed as percentages, and group comparisons were made using the chi-square (x^2^) test or Fisher's exact test. A *p*-value less than 0.05 was considered statistically significant.

## 3 Results

The children were followed up for at least 5 months. As shown in Table 1, the UA-CRPP group included 21 patients, with 12 males and 9 females, and an average age of 4.38 ± 2.09 years. The ORPP group included 36 patients, with 25 males and 11 females, and an average age of 5.36 ± 1.87 years. There were no significant differences between the two groups in terms of gender, age, weight, time from injury to surgery, follow-up time, or injury side. The ORPP group had significantly longer surgery times and hospital stays than the UA-CRPP group (*P* > 0.05), which may be related to the use of a tourniquet during open reduction in the ORPP group.

**Table 1:** Patient demographics.

Variables	UA- CRPP	ORPP	*p*
	*N* = 21	*N* = 36	
Age(years)	4.38 (2.09)	5.36 (1.87)	0.083
Gender:			0.515
Male	12 (57.1%)	25 (69.4%)	
Female	9 (42.9%)	11 (30.6%)	
Weight(kg)	15.0 [13.0;23.8]	20.8 [15.5;25.4]	0.109
Side:			1.000
Right	13 (61.9%)	21 (58.3%)	
Left	8 (38.1%)	15 (41.7%)	
Length of hospital stay(days)	4.76 (0.44)	6.44 (1.05)	< 0.001
From injury to surgery(days)	1.86 (0.57)	2.03 (0.29)	0.215
Operation time(mins)	27.0 [26.0;30.0]	45.0 [42.0;47.5]	< 0.001
Intraoperative blood loss	1.86 (0.36)	1.86 (0.35)	0.968
Follow-up time (weeks)	26.2 (2.79)	27.1 (1.85)	0.174

UA-CRPP, ultrasound combined with x-ray arthrography-guided closed reduction and percutaneous pin fixation group; ORPP, open reduction percutaneous pin fixation group.

As shown in Table 2, there was a significant difference in fracture healing time between the two groups. The healing time in the UA-CRPP group (4.43 ± 0.51 weeks) was shorter than in the ORPP group (5.36 ± 0.54 weeks) (*P* < 0.05). In terms of elbow joint function after surgery, the Flynn elbow function evaluation in the ORPP group showed a 100% excellent rate, with 18 cases classified as excellent, 18 as good, 0 as fair, and 0 as poor. The UA-CRPP group also had a 100% excellent rate, with 9 cases classified as excellent, 12 as good, 0 as fair, and 0 as poor (*P* > 0.05). Postoperative complications in the ORPP group included 1 case of incision infection (1/36, 2.8%), which healed after enhanced dressing changes, and 9 cases of distal humeral Lateral spur formation (9/36, 25.0%). In the UA-CRPP group, there was 1 case of pin site irritation (1/21, 4.8%), which was relieved after pin removal. No cases of distal humeral Lateral spur formation were observed in this group (0/21, 0%). The difference in the incidence of distal humeral Lateral spur formation between the two groups was statistically significant (*P* < 0.05). Both groups did not experience complications such as fish-tail deformity, ischemic necrosis of the fracture site, nonunion, refracture, or iatrogenic nerve injury.

**Table 2:** Radiological and clinical outcomes.

Variables	UA- CRPP	ORPP	*p*
	*N* = 21	*N* = 36	
Radiological union (weeks)	4.43 (0.51)	5.36 (0.54)	< 0.001
Non-union	21 (100%)	36 (100%)	-
Refracture	21 (100%)	36 (100%)	-
Incision infection(Pin tract infection):			1.000
NO	20 (95.2%)	35 (97.2%)	
YES	1 (4.76%)	1 (2.78%)	
Lateral spur formation:			0.019
NO	21 (100%)	27 (75.0%)	
YES	0 (0.00%)	9 (25.0%)	
Flynn:			0.806
Excellent	9 (42.9%)	18 (50.0%)	
Good	12 (57.1%)	18 (50.0%)	
Pass	0	0	
Poor	0	0	
Postoperative nerve injury:	21 (100%)	36 (100%)	-
Avascular necrosis:			1.000
NO	21 (100%)	35 (97.2%)	
YES	0 (0.00%)	1 (2.78%)	

UA-CRPP, ultrasound combined with x-ray arthrography-guided closed reduction and percutaneous pin fixation group; ORPP, open reduction percutaneous pin fixation group.

## 4 Discussion

Humeral epicondyle fractures are common elbow joint fractures in children. In the Jakob classification, type II humeral epicondyle fractures involve the articular surface, making them unstable fractures that typically require surgical treatment (12, 13). However, the choice of treatment method remains a subject of debate (14). This study, through the clinical efficacy analysis of the UA-CRPP and ORPP groups, found that ultrasound-guided, combined with x-ray arthrography-assisted minimally invasive surgery, can achieve good clinical outcomes.

In recent years, ultrasound technology has become an important assessment tool for pediatric fractures due to its advantages of being radiation-free and providing real-time dynamic imaging (15–17). It can clearly visualize the continuity of the cartilage hinge and soft tissue damage, providing guidance for closed reduction (8, 15). In recent years, it has gradually been applied to the treatment of humeral epicondyle fractures (7).

This study demonstrates that the UA-CRPP group, while achieving equally excellent elbow joint function (Flynn score excellent rate of 100%) and maintaining a low overall complication rate, significantly reduced surgery time, hospital stay, and radiological healing time compared to the ORPP group. It also effectively avoided the high incidence of distal humeral Lateral spur formation observed in the ORPP group. In this study, the median surgery time in the UA-CRPP group was significantly shorter than in the ORPP group [27.0 (26.0; 30.0) minutes vs. 45.0 (42.0; 47.5) minutes, *P* < 0.001], and the hospital stay was reduced by 26% (4.76 ± 0.44 days vs. 6.44 ± 1.05 days, *P* < 0.001) **(**Table 1**)**. These results are consistent with other studies (18, 19). Under ultrasound guidance, the closed reduction procedure avoids the extensive exposure, soft tissue dissection, and suturing required in open surgery, significantly simplifying the surgical process and aligning with the principles of Enhanced Recovery After Surgery (ERAS).

This study found that the radiological healing time for fractures in the UA-CRPP group was reduced by 17% compared to the ORPP group (4.43 ± 0.51 weeks vs. 5.36 ± 0.54 weeks, *P* < 0.001) (Table 2). The possible reasons for this include: 1) Maximal Protection of the Fracture Site's Biological Environment: During ORPP surgery, periosteal soft tissue is stripped, disrupting local blood supply. In contrast, the UA-CRPP group used ultrasound guidance for precise operation with percutaneous pinning, which maximized protection of the fracture's blood supply, providing better conditions for callus formation. 2) Precise Reduction for Initial Stability: This study optimized the reduction process through ultrasound guidance (dynamically observing fracture fragment movement and cartilage hinge continuity), followed by x-ray joint imaging to confirm anatomical reduction of the joint surface. This ensured the accuracy of the reduction. Precise reduction itself provides better biomechanical stability, reduces adverse micromovements at the fracture site, and promotes healing. Minimally invasive techniques not only reduce trauma but also optimize the biological basis for healing. In this study, there was no statistically significant difference in the final Flynn elbow function evaluation between the two groups (*P* > 0.05), indicating that despite differences in surgical approach and reduction technique, both methods achieved equally excellent results in restoring elbow function in children.

Studies have shown that the distal humeral Lateral spur formation after ORPP surgery is primarily caused by the stripping of lateral soft tissue (the attachment site of the extensor muscles) during surgery and postoperative reactions (7, 20–22). The results of this study show that 25.0% (9/36) of children in the ORPP group developed distal humeral Lateral spur formation, while no cases occurred in the UA-CRPP group (0/21), with a significant difference (*P* < 0.05). These findings are consistent with some other studies (4, 23). UA-CRPP avoids the lateral incision and extensive soft tissue dissection, thereby maximizing protection of the local blood supply to the epicondyle bone fragment and effectively preventing this complication. Neither group reported long-term deformities such as “fish-tail” deformity or ischemic necrosis of the fracture fragment, but longer-term follow-up is still needed.

This study has several limitations. First, the relatively small sample size, particularly in the UA-CRPP group, may limit the statistical power and generalizability of our findings. Although the preliminary results are encouraging, future studies with larger cohorts are necessary to validate the efficacy and reproducibility of this ultrasound-assisted technique. Second, musculoskeletal ultrasound entails a steep learning curve, requiring systematic training in fundamental knowledge and technical skills before one can become proficient in its application. Furthermore, this study was conducted at a single institution, and it remains uncertain whether this could impact the treatment outcomes. Therefore, multi-center studies are warranted to enhance the scientific rigor and provide further reference for the management of Jakob type II humeral lateral condyle fractures in children.

## 4 Conclusion

In conclusion, this study confirms that UA-CRPP is an effective minimally invasive technique for treating Jakob II humeral epicondyle fractures in children. However, UA-CRPP represents a viable minimally invasive alternative that offers perioperative advantages, rather than demonstrating overall superiority. Future research should involve larger sample sizes, prospective studies, and long-term follow-up, and further investigate the ultrasound learning curve and cost-effectiveness.

## Data Availability

The raw data supporting the conclusions of this article will be made available by the authors, without undue reservation.
